# Perceived Health Needs, Social Support, and Depression Among Patients with Myocardial Infarction: A Cross-Sectional Study

**DOI:** 10.3390/healthcare12242570

**Published:** 2024-12-20

**Authors:** Bushra Alshammari, Fatmah Awad Alrshedy, Awatif M. Alrasheeday, Sameer Alkubati, Mohamed Ayoub Tlili, Wiem Aouicha, Maha Dardouri, Sarah Basheer Alshammari, Hanan Qayyadh Alanazi, Teflah Saud Alshammari, Abdullah Ayad Alharbi, Nashi Masnad Alreshidi, Hind Abdullah Alrashedi, Nouf Shannan Alshammari, Farhan Alshammari, Afrah Madyan Alshammari, Abeer Nuwayfi Alruwaili, Sahar Maziad Alshammari

**Affiliations:** 1 Medical Surgical Nursing Department, College of Nursing, University of Hail, Hail 21424, Saudi Arabia; s.alkubati@uoh.edu.sa; 2 Irada Mental Health Complex, Hail Health Cluster, Hail 21424, Saudi Arabia; falrshdy@moh.gov.sa (F.A.A.); sabaalshammary@moh.gov.sa (S.B.A.); aalharbi49@moh.gov.sa (A.A.A.); hialrshedi@moh.gov.sa (H.A.A.); 3 Nursing Administration Department, College of Nursing, University of Hail, Hail 21424, Saudi Arabia; a.alrasheeday@uoh.edu.sa (A.M.A.); mo.tlili@uoh.edu.sa (M.A.T.); w.aouicha@uoh.edu.sa (W.A.); 4 Department of Nursing, Faculty of Medicine and Health Sciences, Hodeida University, Hodeida P.O. Box 3114, Yemen; 5 Faculty of Medicine of Sousse, University of Sousse, Sousse 4000, Tunisia; m.dardouri@uoh.edu.sa; 6 Department of Maternal and Child Health, College of Nursing, University of Hail, Hail 21424, Saudi Arabia; 7 Sharaf Hospital, Hail Health Cluster, Hail 55471, Saudi Arabia; hqalanizy@moh.gov.sa; 8 College of Nursing, University of Hail, Hail 21424, Saudi Arabia; taflah.alshammary@uoh.edu.sa; 9 Nursing Executive Administration, Hail Health Cluster, Hail 55471, Saudi Arabia; nmalreshidi@moh.gov.sa; 10 Forensic Medical Services Center, Ministry of Health, Hail 55211, Saudi Arabia; noshalshammari@moh.gov.sa; 11 Department of Pharmaceutics, College of Pharmacy, University of Hail, Hail 21424, Saudi Arabia; frh.alshammari@uoh.edu.sa; 12 Department of Maternal and Child Health Nursing, College of Nursing, Jouf University, Sakaka 72388, Saudi Arabia; amshammari@ju.edu.sa; 13 Department of Nursing Administration and Education, College of Nursing, Jouf University, Sakaka 72388, Saudi Arabia; analrwili@ju.edu.sa; 14 School of Nursing and Midwifery, Queen’s University Belfast, Medical Biology Centre, 97 Lisburn Rd, Belfast BT9 7BL, UK

**Keywords:** myocardial infarction, learning needs, depression, social support

## Abstract

Background: Cardiovascular diseases are the leading cause of mortality globally. Myocardial infarction (MI), a major type of cardiovascular disease, presents long-term challenges for patients. Recognizing patients’ perceived health needs and the factors that influence them is crucial for providing comprehensive care and improving outcomes. Aim: This paper explores the perceived health needs, levels of depression, and social support among MI patients, as well as investigates the correlations between these factors. Methods: A cross-sectional study was conducted at King Salman Specialist Hospital from March to June 2024, enrolling 244 MI patients through convenience sampling. Data collection was performed using the following three validated questionnaires: the Cardiac Patient Learning Needs Inventory (CPLNI) to assess the learning needs of MI patients, the Patient Health Questionnaire-9 (PHQ-9) to evaluate depression levels, and the Oslo Social Support Scale (OSSS-3) to measure social support. Statistical analysis was carried out using IBM SPSS Statistics, Version 27. Results: Patients aged 40 years or older and those who were employed exhibited greater learning needs (*p* < 0.001). Female patients were more depressed than males (*p* = 0.008). Higher social support was reported by the female patients, those with a family history of MI, and those who were employed (*p* = 0.002, 0.002, and 0.003, respectively). The total mean score for perceived learning needs was 3.72, with the highest needs in “other pertinent information”, “medication information”, and “anatomy and physiology”. Depression was indicated in 45.1% of MI patients, with significantly higher depression levels in female than in male patients. Additionally, a significant positive correlation was found between social support and perceived learning needs (r = 0.205, *p* = 0.001), as well as a negative correlation between social support and depression (r = −0.441, *p* < 0.001). Conclusions: Addressing both the physical and psychological needs is essential for MI patients. Comprehensive educational programs and mental health support services are necessary for improving outcomes. Personalized patient education and routine depression screenings should be integrated into post-MI care. Future research should examine longitudinal changes in learning needs and mental health status.

## 1. Introduction

Cardiovascular diseases remain the primary cause of mortality globally, taking an estimated 17.9 million lives each year [1]. The World Health Organization aims to achieve a 25% reduction in mortality from noncommunicable diseases, including cardiovascular disease [2].

In fact, recognizing a patient’s perceived health needs is the foundation for providing total care and achieving improved chances. A myocardial infarction (MI) can become a turning point in the patient’s entire health journey, frequently followed by long-term physical, psychological, and social consequences [3].

Despite overcoming the initial medical emergencies related to MI, diagnosed patients continue to face a myriad of challenges. For instance, MI patients often present with a broad range of health issues that can impact their recovery process and overall well-being. These needs can vary depending on personal factors like age, gender, social class, cultural orientation, or health condition, which indicate the multiplicity of patient requirements [4]. One of the main concerns is the patient‘s physical health after having an MI. In fact, post-MI care requires a holistic approach to healthcare, which covers psychological, social, and lifestyle factors including cardiac rehabilitation, medication administration, diet modifications, and symptom treatment [5].

From a psychological perspective, patients might undergo stress, depression, fear of recurrence, and adjustment disorders after they experience a cardiac event [6]. These psychological stressors, however, can severely hinder recoveries, increase cardiovascular risk factors, and may also provide obstacles to medication and treatment adherence [7]. Beyond that, the social and environmental factors represent a determinant factor in the post-MI patients’ perceived health needs. Nowak et al. (2021) noted that problems like social support networks, access to healthcare services, financial constraints, transportation obstacles, and job-related concerns can lead to severe implications for a patient’s knowledge and ability to follow the recommended treatment plans and navigate their recovery journey [8].

Among these factors, depression is particularly concerning due to its pervasive impact on recovery and overall outcomes in MI patients. Studies have established that depression often leads to poor treatment adherence, including reduced compliance with medication regimens, cardiac rehabilitation programs, and lifestyle modifications such as smoking cessation and physical activity [9,10]. Additionally, depression negatively affects quality of life, intensifying fatigue, anxiety, and emotional distress. Physiologically, depression has been linked to heightened inflammatory markers and autonomic nervous system dysregulation, further complicating cardiac recovery. Evidence suggests that depression is an independent predictor of recurrent cardiovascular events and mortality in MI patients, emphasizing the critical need for integrated approaches to address this comorbidity in post-MI care [8].

Admittedly, perceptions of the health needs among patients diagnosed with MI encompass various dimensions, including access to healthcare services, information, and education about their condition, emotional support, rehabilitation programs, and strategies for secondary prevention [11]. Understanding the perceived health needs of patients diagnosed with MI is crucial for optimizing their care and improving outcomes [6,12]. Also, MI is a health condition that requires a comprehensive approach, involving not only healthcare providers but also patients, their families, and community resources for effective management [13,14]. Healthcare professionals are encouraged to employ patient-centered communication strategies to hear the patients’ points of views, preferences, and priorities, which involves them in healthcare-related decision-making [3,10]. By identifying the self-perceived health needs of cardiac patients, healthcare providers can create specific interventions, educational tools, and support systems, leading to the best possible recovery, lower risks of cardiac events, and a better quality of life [15,16]. In addition, collecting views from patients can assist policymakers in shaping such policy strategies as healthcare delivery, resource distribution, and reimbursement models concerning post-MI care improvement. Most importantly, several emerging trends have highlighted the need for comprehensive and proactive approaches to meet MI patients’ perceived health needs. These trends include the rising incidence of MI among the younger populations, the rising prevalence of risk factors such as obesity and sedentary lifestyles, and persistent disparities in healthcare access [17].

In order for providers to enhance patient-centered care delivery, promote informed decision-making, and ultimately improve patient outcomes and the quality of life of MI patients, this study aims to explore the perceived learning needs of patients with MI in King Salman Hospital in Saudi Arabia. Correspondingly, the study’s objectives are as follows:

- Examine the factors associated with the learning needs of patients with MI;
- Assess the levels of depression and social support among patients with MI;
- Investigate the association between sociodemographic factors, learning needs, depression, and social support among patients with MI
- Analyze the correlation between perceived learning needs, depression, and social support in MI patients.

## 2. Materials and Methods

### 2.1. Study Design

A cross-sectional descriptive study was conducted to explore the learning needs of patients with MI at King Salman Specialist Hospital over a four-month period from March to June 2024.

### 2.2. Population and Sampling

The target group included patients diagnosed with MI and receiving the medication and treatments from King Salman Hospital. Participants were enrolled using a convenience sampling method. To ensure adequate power for detecting significant correlations between perceived learning needs, depression, and social support among the MI patients, a minimum sample size of 169 participants was calculated using G*Power software 3.1.9.4. This calculation was based on detecting a medium effect size (r = 0.3) with 80% power and a significance level of 5% (α = 0.05). To enhance the reliability and generalizability of the findings, we enrolled 244 participants, exceeding the minimum required sample size. This larger sample size strengthened the robustness of the statistical analyses and increased the likelihood of identifying meaningful relationships among the study variables.

### 2.3. Sample and Settings

The study was conducted at King Salman Specialist Hospital, a governmental healthcare institution in Hail City. Participants were recruited from the Hail Cardiac Center, a specialized unit within the hospital equipped with over 100 beds and that provides comprehensive care for individuals with cardiovascular conditions in Hail City and its surrounding areas. The center specializes in treating various forms of cardiac diseases across different age groups and offers free healthcare services to Saudi nationals and non-Saudi government employees, thereby improving access to essential cardiac care for a diverse patient population.

Participants included in the study were those diagnosed with MI within the past month, as the need for essential information and the management of illness-related distress is highest immediately post-event and tends to decrease over time [18]. Additional inclusion criteria required participants to be 18 years of age or older, able to read and write, and willing to participate in the study. Exclusion criteria included patients who were unable to complete the questionnaire, as well as those with cognitive impairments, terminal illnesses, or unstable vital signs.

### 2.4. Recruitment Process

The recruitment process involved a collaboration with a gatekeeper—a registered nurse volunteering at the cardiac center. This nurse played a crucial role in facilitating the study by distributing flyers to potential participants. The flyers contained comprehensive information about the study, including its aim, significance, participants requirements, duration of involvement, and the researcher’s contact information. The specific participation requirements were clearly outlined to inform patients of what was expected from them. Additionally, the flyers indicated the time commitment, specifying that the questionnaire would take approximately 5–10 min to complete. Contact information for the researcher was provided for any further inquiries. The questionnaire was administered during the patients’ first follow-up visit, which is typically scheduled two weeks after discharge. Once the patients had agreed to participate, the nurse facilitated contact between the researcher and the participants. The researcher then provided the participants with a consent form and the questionnaire, which was available both in paper format and via an online link, depending on the patients’ preferences. This flexibility allowed participants to choose the format they were most comfortable with.

Participants were given one week to complete the questionnaire. To ensure a higher response rate, a reminder was sent to participants one week after they received the initial questionnaire. Throughout the process, the researcher remained available to answer any questions and offer assistance, ensuring that participants had the necessary support and clarity to complete the questionnaire.

### 2.5. Data Collection

The survey instrument comprised the following four parts:

1. Demographic and Clinical Characteristics: This section included questions on age, gender, marital status, educational level, employment status, and history of MI.
2. Cardiac Patient Learning Need Inventory (CPLNI): The learning needs of patients after acute MI were assessed using the CPLNI, developed by Gerard and Peterson [19]. The original tool consists of 44 items categorized into the following eight domains: introductions to the cardiac care unit, anatomy and physiology of the heart, psychological factors, risk factors, medication information, nutrition information, physical activity, and miscellaneous. Participants ranked their responses on a 5-point Likert scale ranging from “not important” to “very important”. Total scores for CPLNI ranged from 44 to 220, with high scores indicating high demand for information. CPLNI showed strong internal consistency, with an overall Cronbach’s alpha of 0.91. The reliability of individual categories, including anatomy and physiology, psychological factors, risk factors, medication information, diet information, physical activity, and other pertinent information, was also robust, with the coefficient alpha ranging from 0.79 to 0.93 [20]. In line with Gerard and Peterson [19], the content validity of the questionnaire was ensured through expert review, further supporting its applicability in assessing the learning needs of cardiac patients.
3. Patient Health Questionnaire-9 (PHQ-9): The depression levels among MI patients were measured using PHQ-9. The PHQ-9 is a widely used self-administered instrument for assessing depressive symptoms across different populations [21], including nurses [22], and haemodialysis patients [23]. It consists of nine items, each rated on a 4-point Likert scale ranging from 0 (not at all) to 3 (nearly every day), resulting in a total score ranging from 0 to 27. A score of 10 or higher indicates the presence of depressive symptoms. The study utilized the Arabic version of the PHQ-9 to ensure comfort and accuracy for Saudi patients, as it is their preferred language. This version has been validated as a reliable tool for screening depression in the Saudi population [24]. The PHQ-9 questionnaire demonstrated good reliability, with a Cronbach’s alpha of 0.82 [23].
4. Oslo Social Support Scale (OSSS-3): The OSSS-3 has been recommended for epidemiological and population-based surveys due to its simplicity, reliability, and effectiveness in measuring social support. The OSSS-3 was previously employed to evaluate the level of social support among MI patients and has proven its validity in this population [25]. The OSSS-3 scale evaluates social support through three key questions. The first question asks, “How many people are so close to you that you can count on them if you have great personal problems?” Respondents can choose from the following four possible answers: none, 1–2 people, 3–5 people, or 5 or more. The second question measures the level of interest and concern shown by others, with options ranging from none, little, uncertain, some, to a lot. The third question explores how easy it is to receive practical help from neighbors when needed, with responses including very difficult, difficult, possible, easy, and very easy. The first question of the OSSS-3 scale is scored on a 4-point scale, where 1 indicates the lowest level of support (none) and 4 represents the highest level (5 or more people). The second and third questions are scored on a 5-point rating scale, with 1 representing the lowest response (none or very difficult) and 5 indicating the highest level of interest or ease (a lot or very easy). The total OSSS-3 score ranges from 3 to 14, with higher scores indicating greater levels of social support and lower scores indicating weaker levels. Social support levels are categorized into the following three levels: poor social support (scores 3 to 8), moderate social support (scores 9 to 11), and strong social support (scores 12 to 14) [25,26]. Previous studies have conducted the cultural adaptation and validation of these tools for Saudi Arabian patients, including translation from English to Arabic, review, refinement, and pilot testing [27]. The OSSS-3 questionnaire demonstrates high reliability, with a Cronbach’s alpha of 0.925 [27]. Evidence supports the reliability and validity of the OSSS-3 as a measure of the social determinants of health in the general population [25]. These findings confirm that the adapted tools are reliable, valid, and sensitive for use in this population.

### 2.6. Data Analysis

IBM SPSS Statistics, Version 27 (IBM Corp., Armonk, NY, USA), was used to analyze the data. For normally distributed continuous variables, the terms “mean” and “standard deviation” (SD) were utilized, while frequencies and percentages were used to describe categorical variables. The Mann–Whitney or Kruskal–Wallis tests were used to compare the mean scores of the independent and dependent variables. The correlation between perceived learning needs, depression, and social support was examined using Spearman’s correlation coefficient. The significance level was *p* < 0.05.

### 2.7. Ethical Considerations

This study was approved by the University of Hail IRB under the reference number H-2024-103. The participants were informed about the research objectives and assured of the confidentiality of the research data. The survey instrument was administered anonymously, and consent was obtained from all the participants.

## 3. Results

A total of 244 MI patients completed the questionnaire. Table 1 illustrates the socio-demographic characteristics of these patients. A majority of the patients were female (68.0%), with more than one-third aged 40 years or older (Mean = 36.97 ± 12.36). More than half of the patients were employed (53.3%) and less than half had a family history of MI (43.4%). Additionally, a majority the participants had a university-level education (77.9%) and were married (68.9%).

As shown in Table 2, there was a significant correlation between the perceived learning needs of MI patients and their age and employment status, such that patients aged 40 years or old and those who were working had more needs than the other age groups (*p* < 0.001). Regarding depression, the female patients were significantly more depressed than the male patients (*p* = 0.008). The female patients (*p* = 0.002), those who had MI in their families (*p* = 0.002), and those who were not working (*p* = 0.002) had more social support than their counterparts.

**Figure 1:**
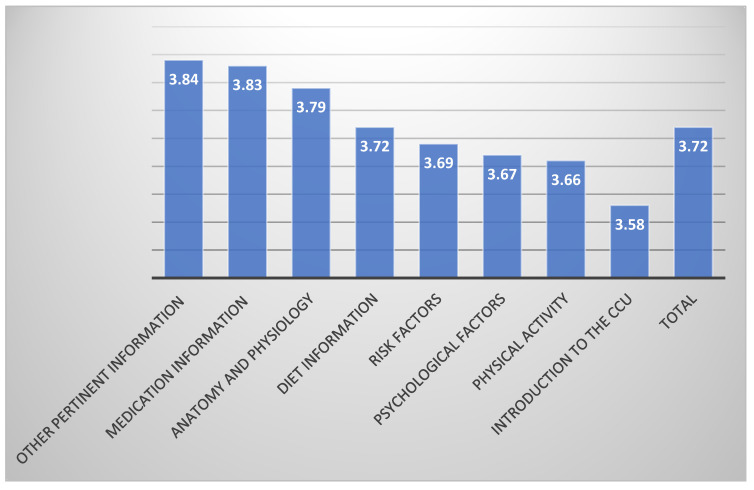
Description of learning needs of MI patients (N = 244).

Figure 1 illustrates the total mean scores of the perceived learning needs of MI patients, along with the mean scores for each subscale. The overall mean score of the perceived learning needs was (M ± SD = 3.72 ± 0.97). The highest needs were in the subscale of “other pertinent information” (M ± SD = 3.84 ± 1.00), which includes topics such as taking a pulse, recognizing angina symptoms, knowing when to call the doctor, understanding post-discharge testing, and learning CPR. This was followed by “medication information” (M ± SD = 3.83 ± 1.09), which covers medication usage, reasons for use, side effects, and problem management, as well as “anatomy and physiology” (M ± SD = 3.79 ± 1.18), which involves understanding chest pain, heart function, heart attack causes, and healing processes.

Table 3 presents the distribution of depression severity among the study participants, as measured by the PHQ-9. The mean depression score was 11.40, with a standard deviation of 7.49. Among the participants, 24.6% had no depression (scores 0–4), 20.5% had mild depression (scores 5–9), 15.6% had moderate depression (scores 10–14), 39.3% had moderately severe depression (scores 15–19), and 24.6% had severe depression (scores 20–27). These findings suggest a significant proportion of the participants experiencing moderate to severe levels of depression, highlighting the need for targeted mental health interventions in this population.

**Figure 2:**
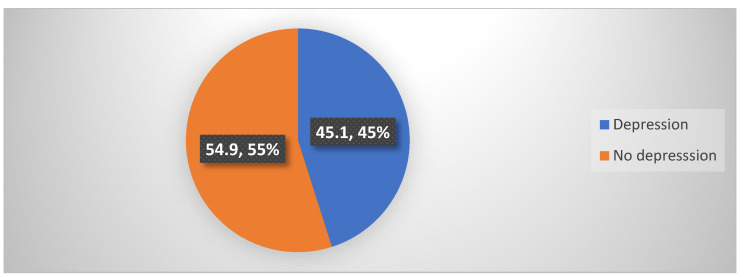
Prevalence of depression among participants (N = 244).

Figure 2 illustrates the prevalence of depression among MI patients. It is shown that less than half of the MI patients had depression (45.1%).

**Figure 3:**
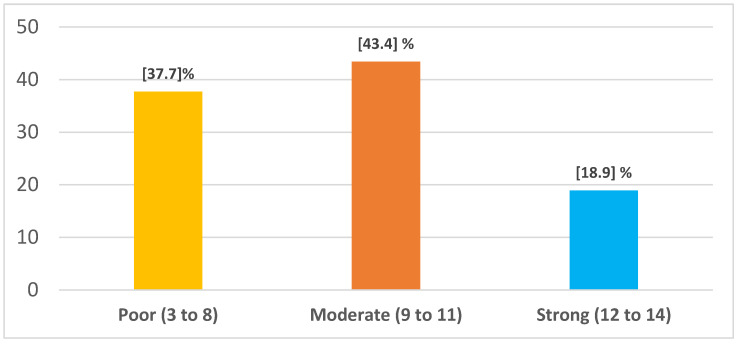
Description of social support for MI patients (N = 244).

Figure 3 illustrates the percentage of social support for MI patients, where the majority of them (43.4%) received moderate social support followed by poor social support (37.7%).

The Pearson correlation analysis (Table 4) shows a significant positive correlation between social support and the perceived learning needs of MI patients (r = 0.205, *p* = 0.001). In addition, a negative correlation was found between social support and depression (r = −441, *p* ≤ 0.001). By contrast, there was no correlation between depression and the patients’ perceived learning needs.

## 4. Discussion

Recognizing the persistent challenges faced by patients following an MI is essential, even after the acute phase has been managed. By identifying and addressing the perceived health needs of these individuals, healthcare providers can establish a foundation for comprehensive care, thereby enhancing the likelihood of improved patient recovery and health outcomes. This study focused on the perceived health needs of patients diagnosed with MI.

The findings revealed that MI patients had significant perceived learning needs, with a total mean score of 3.72 as determined by the CPLNI. These findings align with previous research, such as Arathy et al. (2024) [28] and Huriani (2019) [29], who also identified high learning needs among MI patients. The emphasis on practical skills, medication management, and understanding heart function underscores the necessity for comprehensive education to enhance self-management and clinical outcomes. Healthcare providers should prioritize these educational areas by developing tailored programs to improve patient self-efficacy and long-term health outcomes. Further research should explore learning needs across diverse populations to refine educational strategies.

Among the eight domains, the highest perceived learning needs were identified in the subscale of “other pertinent information”, followed closely by “medication information”, while “anatomy and physiology” information was also highly prioritized.

Our study found that “other pertinent information” is a highly prioritized learning need reported by MI patients. This category includes critical aspects such as taking a pulse, recognizing the signs and symptoms of angina, knowing when to contact a doctor, understanding post-discharge tests, reasons for further testing, and where family members can learn CPR. These findings are similar to those reported by Alsaqri [30]. The importance of this information cannot be overstated, as it empowers patients to manage their condition proactively and respond appropriately to warning signs, potentially preventing further complications or hospitalizations.

The study found that “medication information”, which includes rules about taking medications, reasons for taking them, potential side effects, and what to do if problems arise, is crucial for MI patients. This finding is consistent with the work of Alsaqri (2020) [30]. Understanding this information is essential for adherence to treatment regimens, reducing recurrence risk, and effectively managing side effects [31]. This knowledge fosters patient trust in prescribed therapies, which is vital for long-term health and recovery [18]. Ensuring MI patients receive comprehensive medication information before discharge enables them to manage their health effectively post-discharge.

Lastly, “anatomy and physiology” information, including why chest pain occurs, heart structure, causes of heart attacks, events during a heart attack, and heart healing, was perceived as being highly important. According to Barello et al. [32], this knowledge helps patients to understand their condition, reduces anxiety, and encourages engagement in treatment plans. Well-informed patients are more likely to adhere to lifestyle changes and treatments, which can lead to improved health outcomes [27].

According to Collet et al. [33], the information provided to patients immediately after an MI event, as well as at a time when patients are ready to receive it, is crucial for fostering long-term positive health behaviors. High perceptions of learning needs among MI patients often indicate their readiness for discharge and to return home [34]. To prepare for discharge, it is essential that patients receive comprehensive information from healthcare providers. Addressing the specific learning needs of MI patients enhances their active participation in activities that mitigate the risk of recurrent MI [33,35].

The findings of this study highlight significant variations in the learning needs, depression levels, and social support among MI patients, delineated by age, gender, and employment status. Our study found a significant relationship between the perceived learning needs of MI patients and both their age and employment status. Specifically, patients aged 40 years or older and those who were employed had greater learning needs compared to their younger or unemployed counterparts (*p* < 0.001). Several factors may explain these findings. Older patients likely perceive a higher risk associated with MI and thus feel a greater urgency to understand their condition and management strategies. The increased cardiovascular risks starting from the age of 40 could drive this demographic to seek more information on preventing recurrence and managing their health more effectively. This suggests that older and employed patients may face more complex health management scenarios or have higher expectations for understanding their condition and treatment, necessitating tailored educational interventions to meet these needs effectively.

Employment status also plays a crucial role. Working patients might face additional challenges in balancing their health management with professional responsibilities, leading to a heightened need for information. Understanding symptoms, lifestyle changes, and medication regimens is particularly relevant for this cohort in relation to maintaining their job performance while managing their health. Additionally, being employed might indicate a higher level of daily stress and activity, increasing the demand for knowledge of MI management to mitigate the associated risks.

The results indicate that 45.1% of MI patients experienced depression, corroborating the existing literature that has identified depression as one of the most prevalent psychological reactions post-MI [9]. This significant prevalence underscores the urgent need to address mental health in MI patients, as depression can adversely affect recovery, adherence to treatment, and overall quality of life [36]. Furthermore, the significant negative correlation between social support and depression (r = −0.441, *p* < 0.001) highlights that increased social support is associated with lower levels of depression, reinforcing the protective role of social networks in mental health. These findings emphasize the importance of integrating mental health support and social support systems in the comprehensive care of MI patients.

The gender differences observed in depression levels among MI patients are particularly noteworthy, with female patients exhibiting higher levels of depression than males [37]. This aligns with the existing literature indicating that women often experience higher rates of depression due to a combination of biological, psychosocial, and social factors [38,39,40]. In the specific context of this study, the findings may be further explained by the cultural and societal norms in Saudi Arabia, where women often assume the primary caregiving role within their family. Balancing familial obligations with their own recovery process can add considerable emotional and psychological burdens, which may exacerbate depressive symptoms such as anxiety and fear of recurrence.

Biologically, hormonal fluctuations related to menstruation, pregnancy, and menopause can increase the susceptibility to depression by affecting the neurotransmitter systems involved in mood regulation [41]. Moreover, women may be more likely to report symptoms and seek help for depression [42,43], resulting in higher reported rates. These findings underscore the critical need for comprehensive mental health support in post-MI care, especially for female patients, to address these multifaceted factors contributing to higher depression levels. 

Interestingly, although female MI patients reported higher levels of depression, they also reported higher levels of social support. This finding can be explained by examining the nature and impact of social support in the context of gender-specific experiences. women often maintain stronger social networks and are more likely to seek and receive support from friends, family, and community members [44]. This social integration can provide emotional and practical assistance, which is crucial during the recovery period after an MI [45]. Women are generally more open about their emotional struggles and more proactive in reaching out for help, which can result in higher perceived social support [46].

Those with a family history of MI and those who were employed received higher social support than their counterparts. The increased social support in these groups might be attributed to stronger familial and social networks or workplace interactions that provide emotional and practical assistance. Additionally, individuals with a family history of MI may receive more support because their families are more aware of the condition and its implications, fostering a more supportive environment. Employed individuals might benefit from structured support systems within the workplace, such as employee wellness programs and health benefits, which can offer additional resources and support mechanisms. This finding underscores the need to leverage these support systems in patient education and recovery programs, ensuring that all patients have access to comprehensive support networks.

These findings have important implications for the design of post-MI care programs. Healthcare providers should consider these demographic and psychosocial factors when developing educational materials and support systems. Tailored interventions that address the specific learning needs, mental health concerns, and social support structures of different patient groups could enhance recovery outcomes and improve the overall quality of life for MI patients. Future research should further explore these relationships and develop targeted strategies to optimize patient education and support.

The current study has some limitations. First, because a cross-sectional research design was used, the causal relationship between study variables could not be examined. Second, there was a limitation in the selection of study participants. A convenience sampling procedure was used, thus there was no equal chance of participation for all individuals within the target population, which may introduce bias and limit the generalizability of the findings. Lastly, the study was carried out in a single healthcare institution, and therefore the findings cannot be generalized to patients with MI from other healthcare institutions in Saudi Arabia.

## 5. Study Implications

The effective management of MI patients requires a holistic approach that extends beyond the acute care phase. This study underscores the necessity of addressing both the physical and psychological needs, such as implementing routine depression screenings and integrating targeted mental health support services, which have been shown to reduce psychological distress and improve adherence to treatment plans. Additionally, the development and implementation of tailored educational programs focusing on medication, symptom management, and lifestyle modifications—such as dietary changes and physical activity—during discharge planning are critical for improving long-term recovery outcomes and preventing recurrent cardiac events.

For healthcare practitioners, the findings emphasize the need for personalized patient education tailored to individual learning needs, particularly for older and employed patients, who demonstrated greater demands for information. Clear, accessible guidance on medication regimens, dietary modifications, and risk factor management is essential to enhance self-efficacy and treatment adherence. Additionally, incorporating mental health care, such as cognitive behavioral therapy and support groups, into routine follow-ups is vital for addressing the psychological impact of MI. A comprehensive post-MI care approach that integrates both physical and psychological interventions is necessary to optimize recovery outcomes and prevent recurrent cardiac events. Healthcare systems must prioritize mental health as a core component of cardiovascular care, ensuring patients’ emotional and psychological well-being alongside physical recovery.

Future research should focus on the longitudinal exploration of patients’ evolving needs post-MI, evaluating the effectiveness of various educational and mental health interventions over time. Additionally, socio-cultural factors influencing patients’ learning needs and mental health must be examined to create tailored, culturally sensitive care strategies. Understanding the unique gender differences in depression and social support will be key in developing targeted therapeutic approaches. By addressing these management, practice, and research implications, healthcare providers can significantly improve the quality of care for MI patients, enhancing their overall health outcomes and quality of life.

## 6. Conclusions

The study findings highlight that MI patients, particularly older and employed individuals, demonstrated greater learning needs, with key areas of focus including medication management, recognizing symptoms, and understanding heart function. Additionally, the high prevalence of depression among female patients underscores the need for targeted mental health support services, including routine screening and culturally tailored psychological interventions. The positive correlation between social support and learning needs, as well as the negative correlation between social support and depression, emphasizes the role of strong support systems in recovery.

To improve the outcomes for MI patients, healthcare systems should prioritize the integration of structured educational programs addressing these specific learning needs, alongside routine mental health care. Gender-sensitive interventions should be incorporated into post-MI care, ensuring both physical and psychological recovery, while leveraging social support networks to enhance patient well-being. Future research should explore longitudinal changes in these needs and evaluate the effectiveness of tailored interventions.

## Figures and Tables

**Table 1:** Socio-demographic characteristics of MI patients.

Variables		N	%
Age	Mean ± SD	36.97 ± 12.36	
	29 or less	66	27.0
	30–39	82	33.6
	40 or more	96	39.3
Sex			
	Male	78	32.0
	Female	166	68.0
Do you have MI patient in your family?			
	yes	106	43.4
	no	138	56.6
Employment			
	yes	130	53.3
	no	114	46.7
Education level			
	Secondary	54	22.1
	University	190	77.9
Marital status			
	Single	76	31.1
	Married	168	68.9
Period of disease (days)			
	7–30	102	41.6
	31–60	72	29.4
	61–90	71	29.0

**Table 2:** The relationships between patients’ socio-demographic characteristics and perceived learning needs, depression, and social support.

Variables			CPLNI		PHQ-9		OSS-3	
Age		**n**	**Median (IQR)**	**Mean**	**Median (IQR)**	**Mean**	**Median (IQR)**	**Mean**
	29 or less	66	172.0 (145–187)	128.77	11.0 (4–17)	115.62	8.0 (7–11)	108.47
	30–39	82	135.0 (119–191)	98.60	14.0 (4–17)	118.67	9.0 (8–11)	127.89
	40 or more	96	185.0 (129–206)	138.60	13.0 (5–19)	130.50	9.5 (8–11)	127.54
			<0.001		0.348		0.162	
Sex								
	Male	78	170.0 (129–193)	124.14	8.0 (4–17)	105.17	10.0 (8–12)	142.63
	Female	166	162.0 (129–197)	121.73	14.0 (7–18)	130.64	9.0 (8–10)	113.04
	*p* value		0.803		0.008		0.002	
Do you have MI patient in in your family?								
	yes	106	160.0 (129–197)	119.39	12.0 (4–17)	116.35	10.0 (8–12)	138.29
	no	138	171.0 (129–193)	124.89	12.0 (7–18)	127.22	9.0 (8–10)	110.37
	*p* value		0.546		0.232		0.002	
Employment								
	yes	130	189.0 (129–205)	140.15	12.0 (5–17)	122.68	10.0 (8–11)	135.10
	no	114	152.0 (128–79)	102.38	12.0 (4–18)	122.29	9.0 (7–11)	108.13
	*p* value		<0.001		0.965		0.003	
Education level								
	secondary	54	185.0 (131–202)	135.20	14.0 (0–18)	120.83	9.0 (7–12)	113.46
	University	190	161.0 (129–193)	118.89	12.0 (5–17)	122.97	9.0 (8–11)	125.07
	*p* value		0.134		0.844		0.282	
Marital status								
	single	76	157.0 (135–198)	124.45	13.5 (4–17)	124.37	9.0 (8–12)	122.05
	married	168	171.0 (127–193)	121.62	12.0 (5–18)	121.65	9.0 (8–11)	122.70
	*p* value		0.772		0.780		0.946	
Period of disease (days)								
	7–30	102	171.0 (129–198)	124.09	11.50 (5–17)	119.90	9.00 (8–11)	118.60
	31–60	72	161.0 (129–193)	120.65	14.00 (5–18)	128.05	9.00 (8–11)	122.94
	61–90	71	162.0 (129–193)	122.06	12.00 (5–17)	120.68	9.00 (8–11)	127.67
	*p* value		0.967		0.629		0.851	

Abbreviations: CPLNI: Cardiac Patient Learning Needs Inventory; PHQ-9: Patient Health Questionnaire-9; OSS-3: Social Support Scale; IQR: Interquartile Range.

**Table 3:** Depression levels among participants.

Variables		N	%
**Depression (Mean ± SD) 11.40 ± 7.49**			
	None (0–4)	60	24.6
	Mild (5–9)	50	20.5
	Moderate (10–14)	38	15.6
	Moderately severe (15–19)	96	39.3

**Table 4:** Correlation between perceived learning needs, depression, and social support among MI patients.

Variable		Perceived Learning Needs	Depression
Depression	Correlation Coefficient	0.074	1
	Sig. (2-tailed)	0.250	
Social support	Correlation Coefficient	0.205 **	−0.441 **
	Sig. (2-tailed)	0.001	<0.001

** Correlation is significant at the 0.01 level (2-tailed).

## Data Availability

The data supporting the reported results of this study are available, upon reasonable request, from the corresponding author.

## Abbreviations

MI: Myocardial Infarction, CPLNI: Cardiac Patient Learning Needs Inventory, PHQ-9: Patient Health Questionnaire-9, OSSS-3: Oslo Social Support Scale, IQR: Interquartile Range, SD: Standard Deviation, SPSS: Statistical Package for the Social Sciences

## References

[1] Mendis S., Graham I., Narula J. (2022). Addressing the Global Burden of Cardiovascular Diseases; Need for Scalable and Sustainable Frameworks. Glob. Heart.

[2] World Health Organization (2014). Global Status Report on Noncommunicable Diseases 2014.

[3] Campo G., Tonet E., Chiaranda G., Sella G., Maietti E., Bugani G., Vitali F., Serenelli M., Mazzoni G., Ruggiero R. (2020). Exercise intervention improves quality of life in older adults after myocardial infarction: Randomised clinical trial. Heart.

[4] de Peralta S.S., Dodge K.A., Jones R.A. (2021). An Overview of Quality Improvement Processes and Data Analysis in Perioperative Nursing Practice. AORN J..

[5] Eriksen H., Rautio A., Johnson R., Koepke C., Rink E. (2021). Ethical considerations for community-based participatory research with Sami communities in North Finland. Ambio.

[6] Hennessy M., Dennehy R., Doherty J., O’Donoghue K. (2022). Outsourcing Transcription: Extending Ethical Considerations in Qualitative Research. Qual. Health Res..

[7] Dar T., Radfar A., Abohashem S., Pitman R.K., Tawakol A., Osborne M.T. (2019). Psychosocial Stress and Cardiovascular Disease. Curr. Treat. Options Cardiovasc. Med..

[8] Nowak R.M., Jacobsen G., Limkakeng A., Peacock W.F., Christenson R.H., McCord J., Apple F.S., Singer A.J., deFilippi C.R. (2021). Outpatient versus observation/inpatient management of emergency department patients rapidly ruled-out for acute myocardial infarction: Findings from the HIGH-US study. Am. Heart J..

[9] Alqarni A.S., Pasay-an E., Saguban R., Cabansag D., Gonzales F., Alkubati S., Villareal S., Lagura G.A.L., Alshammari S.A., Aljarboa B.E. (2023). Relationship between the Health Literacy and Self-Medication Behavior of Primary Health Care Clientele in the Hail Region, Saudi Arabia: Implications for Public Health. Eur. J. Investig. Health Psychol. Educ..

[10] Alkubati S.A., Aleyadah H.K., Alboliteeh M., Alharbi A., Alsaif B., Alshammari B., Balawi A. (2024). Predictors to Poor Quality of Life Among Patients with Heart Failure and Its Correlation with Their Medication Adherence: Finding for Healthcare Improvement and Follow-Up. Patient Prefer. Adherence.

[11] Beauchamp A., Talevski J., Niebauer J., Gutenberg J., Kefalianos E., Mayr B., Sareban M., Kulnik S.T. (2022). Health literacy interventions for secondary prevention of coronary artery disease: A scoping review. Open Heart.

[12] Alkubati S.A., Al-Zaru I.M., Khater W., Ammouri A.A. (2013). Perceived learning needs of Yemeni patients after coronary artery bypass graft surgery. J. Clin. Nurs..

[13] Zwack C.C., Smith C., Poulsen V., Raffoul N., Redfern J. (2023). Information Needs and Communication Strategies for People with Coronary Heart Disease: A Scoping Review. Int. J. Environ. Res. Public Health.

[14] Alrasheeday A.M., Alshammari H.S., Alshammari B., Alkubati S.A., Llego J.H., Alshammari A.D., Alshammari M.H., Almohammed R.A., Alsheeb SMs Alshammari F. (2024). Perceived Barriers to Healthy Lifestyle Adherence and Associated Factors Among Patients with Type 2 Diabetes Mellitus: Implications for Improved Self-Care. Patient Prefer. Adherence.

[15] Taylor R.S., Dalal H.M., McDonagh S.T.J. (2022). The role of cardiac rehabilitation in improving cardiovascular outcomes. Nat. Rev. Cardiol..

[16] Alshammari B., Alkubati S.A., Pasay-an E., Alrasheeday A., Alshammari H.B., Asiri S.M., Alshammari S.B., Sayed F., Madkhali N., Laput V. (2023). Sleep Quality and Its Affecting Factors among Hemodialysis Patients: A Multicenter Cross-Sectional Study. Healthcare.

[17] Blakeman J.R., Woith W.M., Astroth K.S., Jenkins S.H., Stapleton S.J. (2022). Women’s Prodromal Myocardial Infarction Symptom Perception, Attribution, and Care Seeking: A Qualitative Multiple Case Study. Dimens. Crit. Care Nurs..

[18] Greco A., Cappelletti E.R., Monzani D., Pancani L., D’Addario M., Magrin M.E., Miglioretti M., Sarini M., Scrignaro M., Vecchio L. (2016). A longitudinal study on the information needs and preferences of patients after an acute coronary syndrome. BMC Fam. Pract..

[19] Gerard P.S., Peterson L.M. (1984). Learning needs of cardiac patients. Cardiovasc. Nurs..

[20] Hamdan F.R.S. (2018). Reliability and Validity of The Arabic Version of Cardiac Patients ’ Learning Needs Inventory (AR-CPLNI ); Among Acute Myocardial Infarction Patients. IOSR J. Nurs. Health Sci..

[21] Costantini L., Pasquarella C., Odone A., Colucci M.E., Costanza A., Serafini G., Aguglia A., Belvederi Murri M., Brakoulias V., Amore M. (2021). Screening for depression in primary care with Patient Health Questionnaire-9 (PHQ-9): A systematic review. J. Affect. Disord..

[22] Pouralizadeh M., Bostani Z., Maroufizadeh S., Ghanbari A., Khoshbakht M., Alavi S.A., Ashrafi S. (2020). Anxiety and depression and the related factors in nurses of Guilan University of Medical Sciences hospitals during COVID-19: A web-based cross-sectional study. Int. J. Afr. Nurs. Sci..

[23] Alkubati S.A., Al-Sayaghi K.M., Salameh B., Halboup A.M., Ahmed W.A.M., Alkuwaisi M.J., Zoromba M.A. (2024). Prevalence of Depression and Its Associated Factors Among Hemodialysis Patients in Hodeida City, Yemen. J. Multidiscip. Healthc..

[24] AlHadi A.N., AlAteeq D.A., Al-Sharif E., Bawazeer H.M., Alanazi H., AlShomrani A.T., Shuqdar R.M., AlOwaybil R. (2017). An arabic translation, reliability, and validation of Patient Health Questionnaire in a Saudi sample. Ann. Gen. Psychiatry.

[25] Kocalevent R.-D., Berg L., Beutel M.E., Hinz A., Zenger M., Härter M., Nater U., Brähler E. (2018). Social support in the general population: Standardization of the Oslo social support scale (OSSS-3). BMC Psychol..

[26] Zhu K., Wang S., Yue Y., Smith B.A., Zhang Z.F., Freudenheim J.L., Niu Z., Zhang J., Smith E., Ye J. (2023). Disparities in insecurity, social support, and family relationships in association with poor mental health among US adults during the COVID-19 pandemic. Sci. Rep..

[27] Alshammari B., Alkubati S.A., Pasay-An E., Alrasheeday A., Madkhali N., Edison J.S., Bakthavatchaalam V., Alshammari M.S., AlRashidi A.A., Alshammari F. (2023). The influence of social support on sleep and fatigue level among patients receiving hemodialysis in Saudi Arabia: A cross-sectional correlational design. Front. Psychol..

[28] Arathy K.S., Moly K.T., Antony R., Vadakkepatt A. (2024). Perceived Health Behaviors and Learning Needs of Patients with Myocardial Infarction. Int. J. Multidiscip. Res..

[29] Huriani E. (2019). Myocardial infarction patients’ learning needs: Perceptions of patients, family members and nurses. Int. J. Nurs. Sci..

[30] Alsaqri S.H., Alkuwaisi M.J., Shafie Z.M., Aldalaykeh M.K., Alboliteeh M. (2020). Saudi myocardial infarction patients’ learning needs: Implications for cardiac education program. Clin. Epidemiol. Glob. Health.

[31] Yu M., Chair S.Y., Chan C.W., Li X., Choi K.C. (2012). Perceived learning needs of patients with heart failure in China: A cross-sectional questionnaire survey. Contemp. Nurse.

[32] Barello S., Graffigna G., Vegni E., Savarese M., Lombardi F., Bosio A.C. (2015). ‘Engage me in taking care of my heart’: A grounded theory study on patient-cardiologist relationship in the hospital management of heart failure. BMJ Open.

[33] Collet J.P., Thiele H., Barbato E., Barthelemy O., Bauersachs J., Bhatt D.L., Dendale P., Dorobantu M., Edvardsen T., Folliguet T. (2021). 2020 ESC Guidelines for the management of acute coronary syndromes in patients presenting without persistent ST-segment elevation. Eur Heart J.

[34] Hydzik P., Kolarczyk E., Kustrzycki W., Kubielas G., Kaluzna-Oleksy M., Szczepanowski R., Uchmanowicz B. (2021). Readiness for Discharge from Hospital after Myocardial Infarction: A Cross-Sectional Study. Int. J. Environ. Res. Public. Health.

[35] Damman P., van ’t Hof A.W., Ten Berg J.M., Jukema J.W., Appelman Y., Liem A.H., de Winter R.J. (2017). 2015 ESC guidelines for the management of acute coronary syndromes in patients presenting without persistent ST-segment elevation: Comments from the Dutch ACS working group. Neth. Heart J..

[36] Dickens C.M., McGowan L., Percival C., Tomenson B., Cotter L., Heagerty A., Creed F.H. (2006). Contribution of depression and anxiety to impaired health-related quality of life following first myocardial infarction. Br. J. Psychiatry.

[37] de Jonge P., Ormel J. (2007). Depression and anxiety after myocardial infarction. Br. J. Psychiatry.

[38] Tang J., Zhang T. (2022). Causes of the male-female ratio of depression based on the psychosocial factors. Front. Psychol..

[39] Hodes G.E., Bangasser D., Sotiropoulos I., Kokras N., Dalla C. (2024). Sex Differences in Stress Response: Classical Mechanisms and Beyond. Curr. Neuropharmacol..

[40] Passarelli M., Casetta L., Rizzi L., Perrella R. (2021). Responses to Stress: Investigating the Role of Gender, Social Relationships, and Touch Avoidance in Italy. Int. J. Environ. Res. Public Health.

[41] Solomon M.B., Herman J.P. (2009). Sex differences in psychopathology: Of gonads, adrenals and mental illness. Physiol. Behav..

[42] Stewart A.L., Kathawalla U.-K., Wolfe A.G., Everson-Rose S.A. (2018). Women’s heart health at mid-life: What is the role of psychosocial stress?. Women’s Midlife Health.

[43] Bogner H.R., Gallo J.J. (2004). Are higher rates of depression in women accounted for by differential symptom reporting?. Soc. Psychiatry Psychiatr. Epidemiol..

[44] Uhing A., Williams J.S., Garacci E., Egede L.E. (2021). Gender differences in the relationship between social support and strain and mortality among a national sample of adults. J. Behav. Med..

[45] Mookadam F., Arthur H.M. (2004). Social support and its relationship to morbidity and mortality after acute myocardial infarction: Systematic overview. Arch. Intern. Med..

[46] Mackenzie C.S., Gekoski W.L., Knox V.J. (2006). Age, gender, and the underutilization of mental health services: The influence of help-seeking attitudes. Aging Ment. Health.

